# A continuum mathematical model of endothelial layer maintenance and senescence

**DOI:** 10.1186/1742-4682-4-30

**Published:** 2007-08-10

**Authors:** Ying Wang, Baltazar D Aguda, Avner Friedman

**Affiliations:** 1 Mathematical Biosciences Institute, and Department of Mathematics, The Ohio State University Columbus, Ohio 43210, USA

## Abstract

**Background:**

The monolayer of endothelial cells (ECs) lining the inner wall of blood vessels deteriorates as a person ages due to a complex interplay of a variety of causes including cell death arising from shear stress of blood flow and cellular oxidative stress, cellular senescence, and decreased rate of replacement of dead ECs by progenitor stem cells.

**Results:**

A continuum mathematical model is developed to describe the dynamics of large EC populations of the endothelium using a system of differential equations for the number densities of cells of different generations starting from endothelial progenitors to senescent cells, as well as the densities of dead cells and the holes created upon clearing dead cells. Aging of cells is manifested in three ways, namely, losing the ability to divide when the Hayflick limit of 50 generations is reached, decreasing replication rate parameters and increasing death rate parameters as cells divide; due to the dependence of these rate parameters on cell generation, the model predicts a narrow distribution of cell densities peaking at a particular cell generation. As the chronological age of a person advances, the peak of the distribution – corresponding to the age of the endothelium – moves towards senescence correspondingly. However, computer simulations also demonstrate that sustained and enhanced stem cell homing can halt the aging process of the endothelium by maintaining a stationary cell density distribution that peaks well before the Hayflick limit. The healing rates of damaged endothelia for young, middle-aged, and old persons are compared and are found to be particularly sensitive to the stem cell homing parameter.

**Conclusion:**

The proposed model describes the aging of the endothelium as being driven by cellular senescence, with a rate that does not necessarily correspond to the chronological aging of a person. It is shown that the age of the endothelium depends sensitively on the homing rates of EC progenitor cells.

## Background

Endothelial cells (EC) form a monolayer called the endothelium which lines the inner wall of blood vessels in the entire circulatory system, from small capillaries to the heart. The endothelium is involved in controlling the passage of materials between tissues and the bloodstream, and its maintenance is of such paramount importance that a blood clotting mechanism – highly conserved in evolution – is immediately elicited when an injury or wound occurs, to plug the damage and stop blood loss (too large a clot, unfortunately, can lead to myocardial infarction or stroke). However, as a person ages many vascular diseases occur due to a variety of causes, including cellular oxidative stress and shear stress of blood flow that induce death of ECs [1-5]. In addition, cellular senescence or aging slows down the replication rates of ECs and, consequently, the rate of repairing endothelium damage. One of the primary causes of cellular aging is the progressive shortening of the ends of chromosomes (telomeres) as cells divide; upon reaching some critical telomere lengths, cells become senescent and lose the ability to proliferate ([6] and the references therein). On the other hand, bone marrow-derived endothelial progenitor cells (EPCs) circulating in the bloodstream can home to holes on the endothelium and differentiate into ECs to provide younger cells [3-8].

In a recent work, Op den Buijs et al. [6] presented a model of endothelium maintenance that incorporates the abovementioned factors. The endothelial wall was modeled as a monolayer of ECs on a square sheet. Computer simulations based on Monte Carlo type [6] approach were carried out involving 500 ECs covering an area of 3.5 × 10^5 ^*μm*^2 ^(the cell radius was taken to be 15 *μm*). The model monitored the changes of the EC monolayer over a period of 60 years, with a discrete time step of 1 year. Dead ECs could be replaced either by division of surrounding ECs or by homing of EPCs according to certain probabilities that are based on experimental evidence. The cells that die are chosen at random at each time step, and when the dead cells are to be replaced by cell division, non-senescent neighboring cells were randomly chosen to divide. It was assumed that the rates of EPC production from the bone marrow and EPC homing are such that there is a steady state number of EPCs in the blood. EPCs that have homed are considered to convert instantly to fully differentiated ECs.

The small number of ECs considered in the computer simulations of Op den Buijs et al. [6] does not allow one to generalize the simulation to larger areas of the vasculature, where hundreds of thousands of ECs are involved. It is of interest to know how the various factors considered in the model of Op den Buijs et al. [6] play out in a large population of ECs, because this information will then allow a correlation of the aging of the organism to the aging of the EC wall. The aim of the present paper is to develop a continuum mathematical model of the EC layer, incorporating maintenance and damaging factors mentioned above. Unlike the discrete model of Op den Buijs et al. [6] the model described in this paper is deterministic. More precisely, the model consists of a dynamical system which describes how densities of EC at different stages of telomere length evolve in time; this evolution is defined by a system of partial differential equations (PDEs).

## Results and discussion

### Model equations

Let *m*_*i*_(*x*, *y*, *t*) be the number density of cells of generation identified by an index *i *that counts the number of mitotic divisions until senescence is reached. The number of cells of generation *i *at time *t *in the area bounded by *x *and *x *+ *dx*, and *y *and *y + dy *is defined to be *m*_*i*_(*x*, *y*, *t*)*dxdy*. For viable cells the index *i *ranges from 0 (for the EPCs that have homed on the endothelial wall) to *N *(for senescent cells). We take *N *= 50 in our simulations (the typical Hayflick limit of human cells). To account for the space created when dead cells are cleared, we introduce the variable called 'hole density', *h*(*x*, *y*, *t*), and define it to be identical to the number density of dead cells before they are cleared.

An important feature of our model is the representation of the observation that a normal EC in contact with other ECs on all sides do not divide (see [6]), and only non-senescent cells that border holes can proliferate. The following rate expression is therefore assumed:

rate of cell division = *λ*_*i*_*m*_*i*_*h*   for 0 ≤ *i *≤ *N *- 1

The proliferation parameter *λ*_*i *_depends on *i *and is expected to decrease with *i *(this dependence is discussed in the next section) – that is, it becomes harder for cells to proliferate as they get older. Also, every viable cell has a probability of dying which we assume to occur at the rate *k*_*i*_*m*_*i *_for 0 ≤ *i *≤ *N*. As with *λ*_*i*_, the death parameter *k*_i _depends on *i *but is expected to increase with *i *(this dependence is discussed in the next section). In the equations below, we only consider the case where viable ECs and EPCs that have homed on the wall do not undergo surface migration – either by diffusion or by transport – on the endothelial wall.

The number density of EPCs evolves according to the following equation

∂m0∂t=γh−λ0m0h−k0m0
 MathType@MTEF@5@5@+=feaafiart1ev1aaatCvAUfKttLearuWrP9MDH5MBPbIqV92AaeXatLxBI9gBaebbnrfifHhDYfgasaacH8akY=wiFfYdH8Gipec8Eeeu0xXdbba9frFj0=OqFfea0dXdd9vqai=hGuQ8kuc9pgc9s8qqaq=dirpe0xb9q8qiLsFr0=vr0=vr0dc8meaabaqaciaacaGaaeqabaqabeGadaaakeaadaWcaaqaaiabekGi2kabd2gaTnaaBaaaleaacqaIWaamaeqaaaGcbaGaeqOaIyRaemiDaqhaaiabg2da9GGaciab=n7aNjabdIgaOjabgkHiTiab=T7aSnaaBaaaleaacqaIWaamaeqaaOGaemyBa02aaSbaaSqaaiabicdaWaqabaGccqWGObaAcqGHsislcqWGRbWAdaWgaaWcbaGaeGimaadabeaakiabd2gaTnaaBaaaleaacqaIWaamaeqaaaaa@4516@

where the first term on the right-hand side is the rate of EPC homing to the wall, and is assumed to be directly proportional to the local hole density; the second term is the rate of cell division, and the last term is the rate of cell death. For the other non-senescent ECs, their number densities change according to

∂mi∂t=2λi−1mi−1h−λimih−kimi(1≤i≤N−1)
 MathType@MTEF@5@5@+=feaafiart1ev1aaatCvAUfKttLearuWrP9MDH5MBPbIqV92AaeXatLxBI9gBaebbnrfifHhDYfgasaacH8akY=wiFfYdH8Gipec8Eeeu0xXdbba9frFj0=OqFfea0dXdd9vqai=hGuQ8kuc9pgc9s8qqaq=dirpe0xb9q8qiLsFr0=vr0=vr0dc8meaabaqaciaacaGaaeqabaqabeGadaaakeaafaqabeqacaaabaWaaSaaaeaacqaHciITcqWGTbqBdaWgaaWcbaGaemyAaKgabeaaaOqaaiabekGi2kabdsha0baacqGH9aqpcqaIYaGmiiGacqWF7oaBdaWgaaWcbaGaemyAaKMaeyOeI0IaeGymaedabeaakiabd2gaTnaaBaaaleaacqWGPbqAcqGHsislcqaIXaqmaeqaaOGaemiAaGMaeyOeI0Iae83UdW2aaSbaaSqaaiabdMgaPbqabaGccqWGTbqBdaWgaaWcbaGaemyAaKgabeaakiabdIgaOjabgkHiTiabdUgaRnaaBaaaleaacqWGPbqAaeqaaOGaemyBa02aaSbaaSqaaiabdMgaPbqabaaakeaacqqGOaakcqqGXaqmcqGHKjYOcqWGPbqAcqGHKjYOcqWGobGtcqGHsislcqaIXaqmcqGGPaqkaaaaaa@5AED@

where the first term is the division rate of cells in the preceding generation, and the last two terms are division and death rates as defined previously.

Since senescent cells do not divide, their evolution equation contains only one source term and a death term, as given below:

∂mN∂t=2λN−1mN−1h−kNmN
 MathType@MTEF@5@5@+=feaafiart1ev1aaatCvAUfKttLearuWrP9MDH5MBPbIqV92AaeXatLxBI9gBaebbnrfifHhDYfgasaacH8akY=wiFfYdH8Gipec8Eeeu0xXdbba9frFj0=OqFfea0dXdd9vqai=hGuQ8kuc9pgc9s8qqaq=dirpe0xb9q8qiLsFr0=vr0=vr0dc8meaabaqaciaacaGaaeqabaqabeGadaaakeaadaWcaaqaaiabekGi2kabd2gaTnaaBaaaleaacqWGobGtaeqaaaGcbaGaeqOaIyRaemiDaqhaaiabg2da9iabikdaYGGaciab=T7aSnaaBaaaleaacqWGobGtcqGHsislcqaIXaqmaeqaaOGaemyBa02aaSbaaSqaaiabd6eaojabgkHiTiabigdaXaqabaGccqWGObaAcqGHsislcqWGRbWAdaWgaaWcbaGaemOta4eabeaakiabd2gaTnaaBaaaleaacqWGobGtaeqaaaaa@46ED@

The number density of dead cells, *q*, is assumed to follow the equation

∂q∂t=D∇2q+∑i=0Nkimi−δq
 MathType@MTEF@5@5@+=feaafiart1ev1aaatCvAUfKttLearuWrP9MDH5MBPbIqV92AaeXatLxBI9gBaebbnrfifHhDYfgasaacH8akY=wiFfYdH8Gipec8Eeeu0xXdbba9frFj0=OqFfea0dXdd9vqai=hGuQ8kuc9pgc9s8qqaq=dirpe0xb9q8qiLsFr0=vr0=vr0dc8meaabaqaciaacaGaaeqabaqabeGadaaakeaadaWcaaqaaiabekGi2kabdghaXbqaaiabekGi2kabdsha0baacqGH9aqpcqWGebarcqGHhis0daahaaWcbeqaaiabikdaYaaakiabdghaXjabgUcaRmaaqahabaGaem4AaS2aaSbaaSqaaiabdMgaPbqabaaabaGaemyAaKMaeyypa0JaeGimaadabaGaemOta4eaniabggHiLdGccqWGTbqBdaWgaaWcbaGaemyAaKgabeaakiabgkHiTGGaciab=r7aKjabdghaXbaa@4A06@

The second term on the right-hand side accounts for the death of all viable cells, and the last term is the rate of clearing dead cells from the endothelial wall. The first term on the right-hand side takes into account that dead cells – prior to being cleared – become loosely attached to the endothelial wall and can diffuse laterally. Note that because of cell-cell adhesion among ECs, no diffusion terms are included in Eqs. (2)–(3). Finally, hole density varies according to

∂h∂t=δq−γh−∑i=0N−1λimih
 MathType@MTEF@5@5@+=feaafiart1ev1aaatCvAUfKttLearuWrP9MDH5MBPbIqV92AaeXatLxBI9gBaebbnrfifHhDYfgasaacH8akY=wiFfYdH8Gipec8Eeeu0xXdbba9frFj0=OqFfea0dXdd9vqai=hGuQ8kuc9pgc9s8qqaq=dirpe0xb9q8qiLsFr0=vr0=vr0dc8meaabaqaciaacaGaaeqabaqabeGadaaakeaadaWcaaqaaiabekGi2kabdIgaObqaaiabekGi2kabdsha0baacqGH9aqpiiGacqWF0oazcqWGXbqCcqGHsislcqWFZoWzcqWGObaAcqGHsisldaaeWbqaaiab=T7aSnaaBaaaleaacqWGPbqAaeqaaaqaaiabdMgaPjabg2da9iabicdaWaqaaiabd6eaojabgkHiTiabigdaXaqdcqGHris5aOGaemyBa02aaSbaaSqaaiabdMgaPbqabaGccqWGObaAaaa@4B55@

where the source term is due to clearing of dead cells, the second term represents homing of EPCs, and the last term corresponds to filling up holes due to divisions of non-senescent cells adjacent to holes.

In all the simulations presented in this paper, we considered a square *R *of 1 cm^2 ^area on the endothelial wall

*R *= {(*x*, *y*)|0 ≤ *x *≤ 1, 0 ≤ *y *≤ 1}

The following set of periodic boundary conditions on the number density of dead cells is assumed:

q(0,y,t)=q(1,y,t),∇q(0,y,t)=∇q(1,y,t)q(x,0,t)=q(x,1,t),∇q(x,0,t)=∇q(x,1,t)
 MathType@MTEF@5@5@+=feaafiart1ev1aaatCvAUfKttLearuWrP9MDH5MBPbIqV92AaeXatLxBI9gBaebbnrfifHhDYfgasaacH8akY=wiFfYdH8Gipec8Eeeu0xXdbba9frFj0=OqFfea0dXdd9vqai=hGuQ8kuc9pgc9s8qqaq=dirpe0xb9q8qiLsFr0=vr0=vr0dc8meaabaqaciaacaGaaeqabaqabeGadaaakeaafaqabeGacaaabaGaemyCaeNaeiikaGIaeGimaaJaeiilaWIaemyEaKNaeiilaWIaemiDaqNaeiykaKIaeyypa0JaemyCaeNaeiikaGIaeGymaeJaeiilaWIaemyEaKNaeiilaWIaemiDaqNaeiykaKIaeeilaWcabaGaey4bIeTaemyCaeNaeiikaGIaeGimaaJaeiilaWIaemyEaKNaeiilaWIaemiDaqNaeiykaKIaeyypa0Jaey4bIeTaemyCaeNaeiikaGIaeGymaeJaeiilaWIaemyEaKNaeiilaWIaemiDaqNaeiykaKcabaGaemyCaeNaeiikaGIaemiEaGNaeiilaWIaeGimaaJaeiilaWIaemiDaqNaeiykaKIaeyypa0JaemyCaeNaeiikaGIaemiEaGNaeiilaWIaeGymaeJaeiilaWIaemiDaqNaeiykaKIaeeilaWcabaGaey4bIeTaemyCaeNaeiikaGIaemiEaGNaeiilaWIaeGimaaJaeiilaWIaemiDaqNaeiykaKIaeyypa0Jaey4bIeTaemyCaeNaeiikaGIaemiEaGNaeiilaWIaeGymaeJaeiilaWIaemiDaqNaeiykaKcaaaaa@7E62@

On a macroscopic scale (which is the view taken in all our simulations), it will be assumed that the ECs and holes are initially distributed so that the total number density is the same at every point, that is,

h(x,y,0)+q(x,y,0)+∑i=0Nmi(x,y,0)=constant,A
 MathType@MTEF@5@5@+=feaafiart1ev1aaatCvAUfKttLearuWrP9MDH5MBPbIqV92AaeXatLxBI9gBaebbnrfifHhDYfgasaacH8akY=wiFfYdH8Gipec8Eeeu0xXdbba9frFj0=OqFfea0dXdd9vqai=hGuQ8kuc9pgc9s8qqaq=dirpe0xb9q8qiLsFr0=vr0=vr0dc8meaabaqaciaacaGaaeqabaqabeGadaaakeaacqWGObaAcqGGOaakcqWG4baEcqGGSaalcqWG5bqEcqGGSaalcqaIWaamcqGGPaqkcqGHRaWkcqWGXbqCcqGGOaakcqWG4baEcqGGSaalcqWG5bqEcqGGSaalcqaIWaamcqGGPaqkcqGHRaWkdaaeWbqaaiabd2gaTnaaBaaaleaacqWGPbqAaeqaaOGaeiikaGIaemiEaGNaeiilaWIaemyEaKNaeiilaWIaeGimaaJaeiykaKcaleaacqWGPbqAcqGH9aqpcqaIWaamaeaacqWGobGta0GaeyyeIuoakiabg2da9iabbogaJjabb+gaVjabb6gaUjabbohaZjabbsha0jabbggaHjabb6gaUjabbsha0jabbYcaSiabdgeabbaa@5EE0@

By dividing both sides of Eq. (8) by *A*, we transform the densities into dimensionless variables. Note that from hereon, *h*, *q*, and *m*_i _(*i *= 1,..., *N*) refer to the dimensionless number densities and that *A *in Eq. (8) is set to 1.

After summing Eqs. (2)–(6), we get

∂∂t(h+q+∑i=0Nmi)=D∇2q
 MathType@MTEF@5@5@+=feaafiart1ev1aaatCvAUfKttLearuWrP9MDH5MBPbIqV92AaeXatLxBI9gBaebbnrfifHhDYfgasaacH8akY=wiFfYdH8Gipec8Eeeu0xXdbba9frFj0=OqFfea0dXdd9vqai=hGuQ8kuc9pgc9s8qqaq=dirpe0xb9q8qiLsFr0=vr0=vr0dc8meaabaqaciaacaGaaeqabaqabeGadaaakeaadaWcaaqaaiabgkGi2cqaaiabgkGi2kabdsha0baacqGGOaakcqWGObaAcqGHRaWkcqWGXbqCcqGHRaWkdaaeWbqaaiabd2gaTnaaBaaaleaacqWGPbqAaeqaaOGaeiykaKcaleaacqWGPbqAcqGH9aqpcqaIWaamaeaacqWGobGta0GaeyyeIuoakiabg2da9iabdseaejabgEGirpaaCaaaleqabaGaeGOmaidaaOGaemyCaehaaa@4718@

and using the divergence theorem with Eq. (7),

∬R∇2q dxdy=∫∂R(∇q⋅n)dS=0
 MathType@MTEF@5@5@+=feaafiart1ev1aaatCvAUfKttLearuWrP9MDH5MBPbIqV92AaeXatLxBI9gBaebbnrfifHhDYfgasaacH8akY=wiFfYdH8Gipec8Eeeu0xXdbba9frFj0=OqFfea0dXdd9vqai=hGuQ8kuc9pgc9s8qqaq=dirpe0xb9q8qiLsFr0=vr0=vr0dc8meaabaqaciaacaGaaeqabaqabeGadaaakeaadaWdsbqaaiabgEGirpaaCaaaleqabaGaeGOmaidaaOGaemyCaehaleaacqWGsbGuaeqaniabgUIiYlabgUIiYdGccqqGGaaicqWGKbazcqWG4baEcqWGKbazcqWG5bqEcqGH9aqpdaWdrbqaaiabcIcaOiabgEGirlabdghaXjabgwSixlabh6gaUjabcMcaPiabdsgaKjabdofatjabg2da9iabicdaWaWcbaGaeyOaIyRaemOuaifabeqdcqGHRiI8aaaa@4F1D@

(where *∂R *is the boundary of *R *and **n **is the vector normal to *∂R*); these equations then imply that

∬R(h+q+∑i=0Nmi)dxdy=constant,Afor all t>0
 MathType@MTEF@5@5@+=feaafiart1ev1aaatCvAUfKttLearuWrP9MDH5MBPbIqV92AaeXatLxBI9gBaebbnrfifHhDYfgasaacH8akY=wiFfYdH8Gipec8Eeeu0xXdbba9frFj0=OqFfea0dXdd9vqai=hGuQ8kuc9pgc9s8qqaq=dirpe0xb9q8qiLsFr0=vr0=vr0dc8meaabaqaciaacaGaaeqabaqabeGadaaakeaafaqabeqacaaabaWaa8GuaeaacqGGOaakcqWGObaAcqGHRaWkcqWGXbqCcqGHRaWkdaaeWbqaaiabd2gaTnaaBaaaleaacqWGPbqAaeqaaaqaaiabdMgaPjabg2da9iabicdaWaqaaiabd6eaobqdcqGHris5aOGaeiykaKIaemizaqMaemiEaGNaemizaqMaemyEaKhaleaacqWGsbGuaeqaniabgUIiYlabgUIiYdGccqGH9aqpcqqGJbWycqqGVbWBcqqGUbGBcqqGZbWCcqqG0baDcqqGHbqycqqGUbGBcqqG0baDcqqGSaalcqWGbbqqaeaacqqGMbGzcqqGVbWBcqqGYbGCcqqGGaaicqqGHbqycqqGSbaBcqqGSbaBcqqGGaaicqWG0baDcqGH+aGpcqaIWaamaaaaaa@6290@

Since one EC covers an area of the order of 300 square microns, *A *is in the order of 0.3 × 10^6 ^cells per cm^2 ^area. In our simulations, we scale all the densities such that the constant *A *is set to 1. Note that if *h*(*x*, *y*, 0), *q*(*x*, *y*, 0) and *m*_i_(*x*, *y*, 0) (*i *= 1,..., *N*) are all constants, then the system (2)–(6) becomes a system of ordinary differential equations.

**Figure 1:**
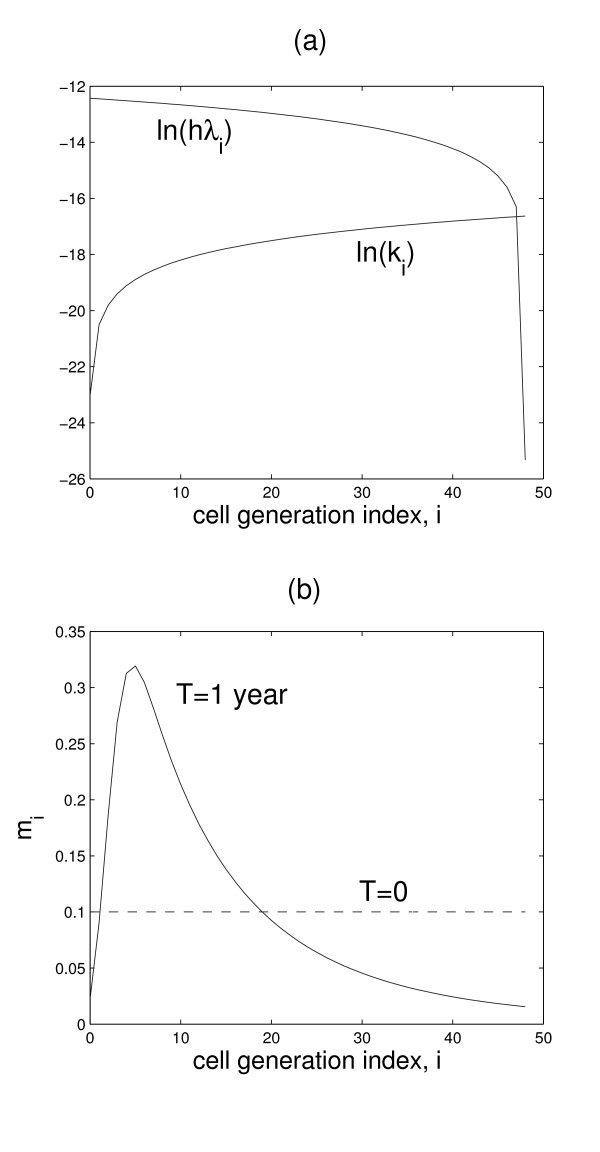
**Cell rate parameters and generation profiles**. (a) Variations of cell replication rate coefficients (*hλ*_*i*_) and death rate coefficients (*k*_*i*_) with cell generation. (b) Figure demonstrating that due to the variations in (a), a sharply peaked distribution (black solid curve) is spontaneously generated, even from an initially flat distribution (dashed line).

Table 1 is a summary of the parameters used in the computer simulations. The estimation of these parameters is discussed under the section *Methods*. Figure 1(a) shows the assumed dependence of the rate coefficients for cell replication and death.

**Table 1:** Parameter values used in the computer simulations. (Note that the dimension of *λ*_i_, (i = 0, 1,..., N) is cm^-2 ^s^-1^, but it becomes s^-1 ^when dimensioness number densities are used).

Parameter	Value	Description
N	50	number of generations from progenitor to senescence
D	10^-8 ^cm^2 ^s^-1^	diffusion coefficient of dead cells
*k*_0_	0 s^-1^	death rate coefficient of progenitor cells
*k*_*N*_	0.6 × 10^-7 ^s^-1^	death rate coefficient of senescent cells
*k*_*i *_(*i *= 1,..., *N *- 1)	(k_N _+ 1)^i/N ^- 1 s^-1^	death rate coefficient of cells of age i
*λ*_0_	0.4 × 10^-4 ^s^-1^	division rate constant of progenitor cells
*λ*_*N*_	0 s^-1^	division rate constant of senescent cells
*λ*_*i *_(*i *= 1,..., *N *- 1)	(*λ*_0 _+ 1)^(N-i)/N ^- 1 s^-1^	division rate constant of cells at age *i*
*γ*	10^-9 ^- 10^-8 ^day^-1 ^≈ (10^-9 ^- 10^-8^) × 10^-5 ^s^-1^	homing rate coefficient of progenitor cells
*δ*	6 × 10^-7 ^s^-1^	clearance rate coefficient of dead cells

### Cell generation profiles and aging of the endothelium

**Figure 2:**
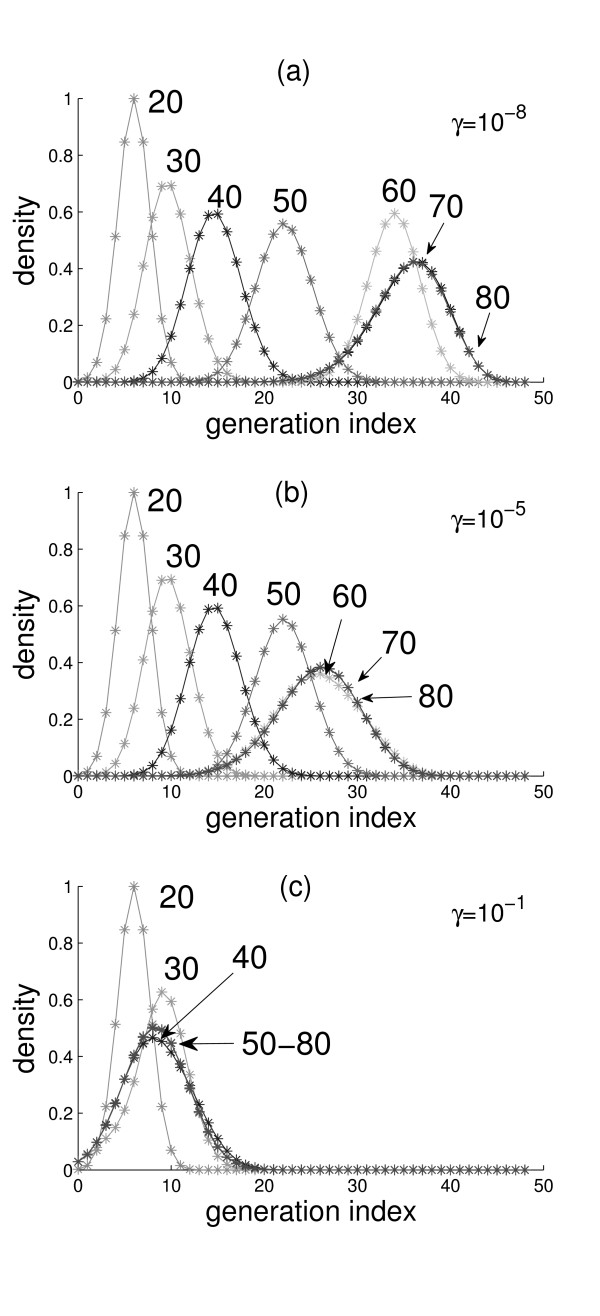
**Cell generation profiles**. Cell generation profiles for a person with advancing chronological age of *T *= 20, 30, 40, 50, 60, 70, and 80 years (numbers near the peaks of the curves). The three values used in the simulations for the progenitor cell homing parameter *γ *are indicated; values of all other parameters are in Table 1.

In this section we take *h*(*x*, *y*, 0), *q*(*x*, *y*, 0) and *m*_i_(*x*, *y*, 0) (*i *= 1,..., *N*) to be constants – that is, these dimensionless number densities are homogeneous in space. We refer to the distribution *G*(*t*) = {*m*_i_(*t*)} as the 'cell generation profile' of the endothelium at time *t*. It is assumed that, on average, the endothelium of an individual at any age is composed of a distribution *G *of finite spread. Interestingly, due to the dependence of the cell division and death rate constants (*λ*_i _and k_i_) on cell generation *i *(see Fig. 1a), our model spontaneously generates *G*(*t*) profiles that look like Gaussian distributions (see Fig. 2) – that is, even an initially flat distribution eventually becomes Gaussian-like given enough time, as shown in Fig. 1b (an initial flat distribution was merely used for numerical reasons and does not represent any biological condition). In our simulations, the chronological age of an individual is measured by the independent variable *t *in the dynamical Eqs. (2)–(6). If we take the peak of the distribution *G *as a measure of the endothelium's age, the model simulations presented below demonstrate that the endothelium does not have to age at the same rate as the individual's chronological age. We investigated the progression of *G*(*t*) from *t *= 20 years to *t *= 80 years – that is, the aging of the endothelium of a 20-year old person in 60 years. Currently, there are no published measurements that permit us to correlate an individual's chronological age to the cell generation index i* where *G *peaks; for our initial condition, we arbitrarily assumed that a 20-year old has a *G *that peaks at i* = 6 (see leftmost curve in Fig. 2).

Because the initial conditions *h*(*x*, *y*, 0), *q*(*x*, *y*, 0) and *m*_*i*_(*x*, *y*, 0) (*i *= 0, 1, ..., *N*) are constants, the simulations shown in Fig. 2 are the solutions of the system of ordinary differential Eqs. (2)–(5). Since cells divide only if they are adjacent to a hole (see Eq. (1)), *h*(*x*, *y*, 0) must be nonzero for *G*(*t*) to evolve in time; we call *h*(*x*, *y*, 0) (also symbolized by *h*_0_) the background hole density that is always present and drives the normal aging process. As shown in Fig. 2(a), *G *moves toward senescence at variable rates: initially slow from 20 to 50 years, followed by higher rates for a few decades, and then a final slowing down towards a stationary profile (the overlapping peaks at i* = 36). Note that *G *broadens as i* increases. The total number of cells, (q+∑i=0Nmi)
 MathType@MTEF@5@5@+=feaafiart1ev1aaatCvAUfKttLearuWrP9MDH5MBPbIqV92AaeXatLxBI9gBaebbnrfifHhDYfgasaacH8akY=wiFfYdH8Gipec8Eeeu0xXdbba9frFj0=OqFfea0dXdd9vqai=hGuQ8kuc9pgc9s8qqaq=dirpe0xb9q8qiLsFr0=vr0=vr0dc8meaabaqaciaacaGaaeqabaqabeGadaaakeaacqGGOaakcqWGXbqCcqGHRaWkdaaeWaqaaiabd2gaTnaaBaaaleaacqWGPbqAaeqaaaqaaiabdMgaPjabg2da9iabicdaWaqaaiabd6eaobqdcqGHris5aOGaeiykaKcaaa@3A0A@, is monitored during all the simulations; the total remains approximately constant until about age 50, when a slow rate of decline begins. At the advanced age of 80 years, a significant rise in the number of dead cells is observed.

The speed by which *G *moves towards senescence depends sensitively on the value of the EPC homing parameter *γ*. This parameter was varied between 0 and 1, and we found that *G*(*t*) becomes slower if *γ *is increased, and faster if *γ *is decreased. For *γ *= 10^-9^, the results are very similar to those of Fig. 2(a). For large values of *γ *(>10^-7^), some interesting results can be observed. When *γ *= 10^-5^, a stationary *G *is reached with i* = 27 at the age of *t *= 60 (see Fig. 2(b)). For *γ *= 10^-1 ^(see Fig. 2(c)), *G *moves forward (increasing i*) for some time and then *back *to a stationary *G *with i* = 9; clearly, this high value of *γ *is unrealistic as one does not expect the peak i* of the EC aging profile to become "younger" as a person ages. These results suggest that for an average person, *γ *ranges from 10^-9 ^to 10^-8 ^s^-1^.

### Healing of damaged endothelium

**Figure 3:**
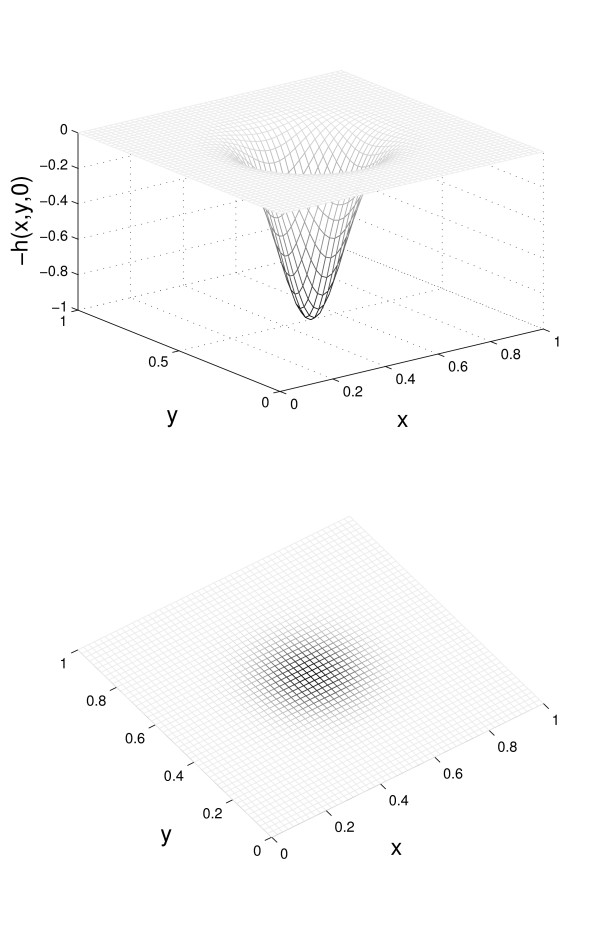
**Initial hole density profile**. The initial hole densities *h*(*x*, *y*, 0) of the damaged endothelium (see Eqn. (10)). The bottom figure shows the two-dimensional endothelium with hole densities proportional to the darkness of the regions.

Here, we are interested in the dynamics of spatially nonhomogenous hole densities much larger than the background *h*_0 _considered in the previous section (and we set *h*_0 _= 0 in the discussion below). In general, the expansion or shrinkage of holes depends on the parameters *γ*, *δ*, *λ*_i _and *k*_i _(*i *= 0, 1,..., *N*). We used our model to study the rates of filling up holes in the endothelia of persons of different ages. We start with the spatial profile of the holes defined by Eq. (10); this profile is plotted in Figure 3.

*h*(*x*, *y*, 0) = exp [-50((*x *- 0.5)^2 ^+ (*y *- 0.5)^2^)]

To determine the initial cell densities {*q*(*x*, *y*, 0), *m*_i_(*x*, *y*, 0)}, when there is a hole given by Eq. (10) at *t *= 0, the profile *G*(*T*) (for *T *= 20, 40, or 60 years) is first determined as in the previous section (i.e., with only the nonzero background hole density *h*_0_). Let {*m*_i_^*T*^} be the set of cell densities for the *G*(*T*), and *q*^*T *^be the dead-cell densities at the same time *T*. In order to keep the total cell density constant (according to Eq. (8)), we set

*m*_*i*_(*x*, *y*, 0) = *α*(*x*, *y*)*m*_*i*_^*T*^   for all *m*_*i*_^*T *^∈ *G*(*T*)

*q*(*x*, *y*, 0) = *α*(*x*, *y*)*q*^*T*^

where

α(x,y)=(A−h(x,y,0))(qT+∑i=1NmiT)
 MathType@MTEF@5@5@+=feaafiart1ev1aaatCvAUfKttLearuWrP9MDH5MBPbIqV92AaeXatLxBI9gBamXvP5wqSXMqHnxAJn0BKvguHDwzZbqegyvzYrwyUfgarqqtubsr4rNCHbGeaGqiA8vkIkVAFgIELiFeLkFeLk=iY=Hhbbf9v8qqaqFr0xc9pk0xbba9q8WqFfeaY=biLkVcLq=JHqVepeea0=as0db9vqpepesP0xe9Fve9Fve9GapdbaqaaeGacaGaaiaabeqaamqadiabaaGcbaacciGae8xSdeMaeiikaGIaemiEaGNaeiilaWIaemyEaKNaeiykaKIaeyypa0ZaaSaaaeaacqGGOaakcqWGbbqqcqGHsislcqWGObaAcqGGOaakcqWG4baEcqGGSaalcqWG5bqEcqGGSaalcqaIWaamcqGGPaqkcqGGPaqkaeaacqGGOaakcqWGXbqCdaahaaWcbeqaaiabdsfaubaakiabgUcaRmaaqahabaGaemyBa02aa0baaSqaaiabdMgaPbqaaiabdsfaubaakiabcMcaPaWcbaGaemyAaKMaeyypa0JaeGymaedabaGaemOta4eaniabggHiLdaaaaaa@61C9@

with *A *set to 1. Note that *α *depends both on space and the chronological age *T *of the person. The dynamics of hole filling by ECs is then simulated by solving Eqs. (2)–(8), using the initial cell density and hole profiles given by Eq. (11) and Eq. (10), respectively.

Let *θ *(*t*) be the fraction of holes remaining (in excess of the background *h*_0_) at time *t*; this fraction is defined by

∬R(h(x,y,t)−h0) dx dy=θ(t) ∬R(h(x,y,0)−h0)dx dy
 MathType@MTEF@5@5@+=feaafiart1ev1aaatCvAUfKttLearuWrP9MDH5MBPbIqV92AaeXatLxBI9gBaebbnrfifHhDYfgasaacH8akY=wiFfYdH8Gipec8Eeeu0xXdbba9frFj0=OqFfea0dXdd9vqai=hGuQ8kuc9pgc9s8qqaq=dirpe0xb9q8qiLsFr0=vr0=vr0dc8meaabaqaciaacaGaaeqabaqabeGadaaakeaadaWdsbqaaiabcIcaOiabdIgaOjabcIcaOiabdIha4jabcYcaSiabdMha5jabcYcaSiabdsha0jabcMcaPiabgkHiTiabdIgaOnaaBaaaleaacqaIWaamaeqaaOGaeeykaKIaeeiiaaIaemizaqMaemiEaGNaeeiiaaIaemizaqMaemyEaKhaleaacqWGsbGuaeqaniabgUIiYlabgUIiYdGccqGH9aqpiiGacqWF4oqCcqGGOaakcqWG0baDcqGGPaqkcqqGGaaidaWdsbqaaiabcIcaOiabdIgaOjabcIcaOiabdIha4jabcYcaSiabdMha5jabcYcaSiabicdaWiabcMcaPiabgkHiTiabdIgaOnaaBaaaleaacqaIWaamaeqaaOGaeiykaKIaemizaqMaemiEaGNaeeiiaaIaemizaqMaemyEaKhaleaacqWGsbGuaeqaniabgUIiYlabgUIiYdaaaa@677E@

**Figure 4:**
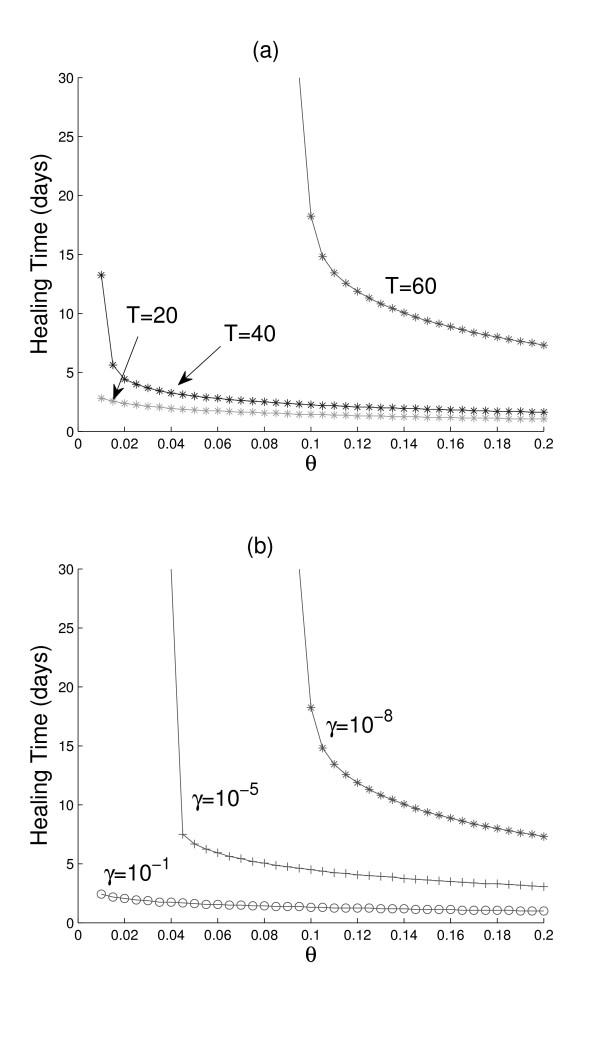
**Healing times of damaged endothelium**. (a) Starting with identical hole density profiles (shown in Fig. 3) on their endothelia, the time it takes (healing time) for the hole to be reduced to a fraction *θ *is shown for a young (*T *= 20 years), a middle-aged (*T *= 40), and an old (*T *= 60) person. Parameter values as in Table 1, and *γ *= 10^-8^. (b) Starting with the cell generation profile of an old person (*T *= 60 years) with a damaged endothelium (hole density profile in Fig. 3), the healing times are determined for the three *γ *values indicated. Other parameter values in Table 1.

Figure 4a shows a comparison of the times required by the three age groups to reduce the hole fraction by 80% or more (*θ *≤ 0.2). Eighty percent of the hole (*θ *= 0.2) is filled in a day for a 20-year old person, almost 2 days for a 40-year old, and about a week for a 60-year-old. Note that a 60-year-old person requires more than one month to achieve 90% recovery (*θ *= 0.1), and healing times increase very rapidly for higher recoveries. The above conclusions are drawn under the assumption that *γ *is the same for all ages (*γ *= 10^-8^). However, we expect *γ *to decrease with age, implying that the recovery of an older person will be even slower.

Figure 4b demonstrates the sensitivity of healing durations on the parameter *γ*. An interesting result shown in this figure is that, despite the reduced replication rates and increased death rates of the ECs of a 60-year old person, if somehow *γ *is increased from 10^-8 ^to 10^-1^, the possibility exists that the old person's endothelium can heal as fast as that of a 20-year old (see also Fig. 2c for the possible reversal of endothelium aging at increased *γ*). This result is not surprising at all when one remembers that increasing *γ *means larger homing rates of EPCs (whose cell generation index *i *is 0), a situation akin to transplanting patches of brand new ECs to an old endothelium.

Our model of healing of a wounded endothelium has the limitation that it only involves ECs; in reality, the healing process is very complex involving many other types of cells, including platelet cells that aggregate on the wound during the clotting cascade. Recent work [5] has shown that, indeed, platelets may be involved in the recruitment of EPCs as well as in the stimulation of proliferation and migration of non-senescent ECs surrounding the wound. Since the clotting process is fast in relation to the proliferative phase of wound healing considered in this paper, our results may still hold even if platelet aggregation is included in the model.

## Conclusion

We developed a mathematical model of the endothelium to investigate the interactions of maintenance factors such as cell division and stem cell homing, and damaging factors such as cellular senescence and death. Our model involves a system of differential equations that offers a deterministic view of the average dynamics of large EC populations, hence, providing predictions that apply to large areas of the vasculature – in contrast to the computational model of Op den Buijs et al. [6] which focuses on a few hundreds of ECs.

Our model introduced some novel features. The model explicitly accounts for the number densities of cell generations starting from endothelial progenitors to senescent cells, in addition to the densities of dead cells and the holes created upon clearing these cells. Aging of cells is represented by three mechanisms, namely, losing the ability to divide when the Hayflick limit of 50 generations is reached, decreasing replication rate parameters and increasing death rate parameters as cells divide. These features implement direct links between aging at the cellular level and aging of a person. Due to the dependence of the cell division and death rate parameters on cell generation, our model predicts a narrow distribution of cell densities peaking at a particular cell generation – it is this peak that characterizes the age of the endothelium. As the chronological age of a person advances, the peak of the distribution moves towards senescence correspondingly. Our computer simulations demonstrate that sustained and enhanced stem cell homing can halt the aging process of the endothelium by maintaining a stationary cell density distribution that peaks well before the Hayflick limit. The healing rates of damaged endothelia for young, middle-aged, and old persons are compared and are found to be particularly sensitive to the stem cell homing parameter.

In conclusion, our model predicts that the age of an endothelium corresponds to a Gaussian-like distribution of EC generations with a distribution peak that moves towards senescence at a rate that can be manipulated by varying the rate parameters of cellular processes that are considered in our model. The model also suggests that stem cell transplantation may enhance the rate of endothelial replacement, so that the effect of aging could be reversed. The theoretical model presented in this paper has obvious limitations; for example, aging is also associated with progressive thickening of arteries (atherosclerosis) and plaque formation in the endothelium – a complex process that is not considered here.

## Methods

### Parameter estimation

The model parameters listed in Table 1 are determined as follows. Based on published values for EC migration in cultures containing angiogenic factors (Stokes et al. [9]), we estimate the diffusion coefficient of dead ECs to be in the order of 10^-8 ^cm^2 ^s^-1^. We assume that cell death rate coefficients range from a minimum of zero (for *k*_0_) to a maximum value of 6 × 10^-8 ^s^-1 ^(for *k*_*N*_), with intervening values given by the equation *k*_*i *_= (*k*_*N *_+ 1)^*i*/*N *^- 1 (see Fig. 1a). The results remain essentially the same if *k*_*i *_is interpolated in different ways (e.g. linearly) between *k*_0 _and *k*_*N*_.

According to [6], ECs *in vivo *replicate at a rate of 0.1% per day, which corresponds to a division rate constant of 10^-3 ^day^-1 ^or 10^-8 ^s^-1 ^– this value will be used for the estimate of average cell replication rate constants for the entire cell population, ranging from zero (*λ*_*N *_= 0 for senescent cells) to a maximum of *λ *_0 _(= 4 × 10^-5 ^s^-1 ^for progenitor cells), and with intermediate values determined from the equation *λ*_*i *_= (*λ*_0 _+ 1)^(*N*-*i*)/*N *^- 1 (see Fig. 1a). The results remain essentially the same if *λ*_*i *_is interpolated in different ways between *λ*_0 _and *λ*_*N*_.

To understand the time scale of the cell replication dynamics of an entire EC population using the above parameter values, first consider the equation for the reproduction of one type of cell only, that is, dmidt=(λih)mi
 MathType@MTEF@5@5@+=feaafiart1ev1aaatCvAUfKttLearuWrP9MDH5MBPbIqV92AaeXatLxBI9gBaebbnrfifHhDYfgasaacH8akY=wiFfYdH8Gipec8Eeeu0xXdbba9frFj0=OqFfea0dXdd9vqai=hGuQ8kuc9pgc9s8qqaq=dirpe0xb9q8qiLsFr0=vr0=vr0dc8meaabaqaciaacaGaaeqabaqabeGadaaakeaadaWcaaqaaiabdsgaKjabd2gaTnaaBaaaleaacqWGPbqAaeqaaaGcbaGaemizaqMaemiDaqhaaiabg2da9iabcIcaOGGaciab=T7aSnaaBaaaleaacqWGPbqAaeqaaOGaemiAaGMaeiykaKIaemyBa02aaSbaaSqaaiabdMgaPbqabaaaaa@3E0A@. The time it takes for this cell population to double is given by Ti=ln⁡2λih
 MathType@MTEF@5@5@+=feaafiart1ev1aaatCvAUfKttLearuWrP9MDH5MBPbIqV92AaeXatLxBI9gBaebbnrfifHhDYfgasaacH8akY=wiFfYdH8Gipec8Eeeu0xXdbba9frFj0=OqFfea0dXdd9vqai=hGuQ8kuc9pgc9s8qqaq=dirpe0xb9q8qiLsFr0=vr0=vr0dc8meaabaqaciaacaGaaeqabaqabeGadaaakeaacqWGubavdaWgaaWcbaGaemyAaKgabeaakiabg2da9maalaaabaGagiiBaWMaeiOBa4MaeGOmaidabaacciGae83UdW2aaSbaaSqaaiabdMgaPbqabaGccqWGObaAaaaaaa@38E1@. For *h *= 0.1 (see Results and Discussion), ∑i=0NTi≈430
 MathType@MTEF@5@5@+=feaafiart1ev1aaatCvAUfKttLearuWrP9MDH5MBPbIqV92AaeXatLxBI9gBaebbnrfifHhDYfgasaacH8akY=wiFfYdH8Gipec8Eeeu0xXdbba9frFj0=OqFfea0dXdd9vqai=hGuQ8kuc9pgc9s8qqaq=dirpe0xb9q8qiLsFr0=vr0=vr0dc8meaabaqaciaacaGaaeqabaqabeGadaaakeaadaaeWbqaaiabdsfaunaaBaaaleaacqWGPbqAaeqaaaqaaiabdMgaPjabg2da9iabicdaWaqaaiabd6eaobqdcqGHris5aOGaeyisISRaeGinaqJaeG4mamJaeGimaadaaa@3AA2@ days or 14 months for *N *= 50; thus, this choice of *N *corresponds to a cell life span from progenitor to senescence of approximately 14 months. Of course, this short time span for cellular senescence is different from the much slower aging rate of the endothelium (discussed under *Results and Discussion*) because of contact inhibition between EC cells and replenishment of holes by EPC homing.

The value chosen for the clearing rate coefficient, *δ*, of dead cells is based on the assumption that it is an order of magnitude larger than *k*_*N*_, the death rate constant for senescent cells. Lastly, the value of the EPC homing rate coefficient, *γ*, was determined to range from 10^-9 ^to 10^-8 ^s^-1 ^by imposing biological constraints on the results of the model simulations (see section on *Results and Discussion*).

### Computational method

The ODEs for calculating cell generation profiles were integrated using the stiff solver *ode15s *in MATLAB 7.4.0. The PDEs describing the healing of damaged endothelium were solved using C++. Spatial discretization was carried out using finite difference method with periodic boundary conditions. All plots were made using MATLAB 7.4.0.

## Competing interests

The author(s) declare that they have no competing interests.

## Authors' contributions

YW performed the numerical simulations. BDA and AF formulated the model equations, wrote the manuscript, and jointly supervised YW's work. All authors read and approved the final manuscript.
